# Ginger (*Zingiber officinale*) dietary supplementation in mice regulates liver antioxidant defense systems in a dose- and age-dependent

**DOI:** 10.3389/fphar.2025.1597599

**Published:** 2025-05-15

**Authors:** Maima Matin, Kamil Wysocki, Jarosław Olav Horbańczuk, Luciana Rossi, Atanas G. Atanasov

**Affiliations:** ^1^ Institute of Genetics and Animal Biotechnology of the Polish Academy of Sciences, Warsaw, Poland; ^2^ Department of Veterinary Medicine and Animal Sciences – DIVAS, University of Milan, Milan, Italy; ^3^ Ludwig Boltzmann Institute Digital Health and Patient Safety, Medical University of Vienna, Vienna, Austria

**Keywords:** bioactivity, antioxidant, hepatoprotection, ginger, liver, aging, metabolism

## Abstract

**Introduction:**

Oxidative stress and impaired antioxidant defenses contribute significantly to liver dysfunction, particularly with aging. This study evaluated the dose- and age-dependent effects of dietary ginger (*Zingiber officinale*) supplementation on liver antioxidant defense systems in mice.

**Methods:**

Male Swiss Webster mice aged 3, 6, and 12 months (n = 48 per age group) received standard feed or feed supplemented with either 0.6% or 1.8% dried ginger powder for 3 months. Liver tissue was analyzed for multiple antioxidant parameters, including DPPH radical scavenging activity, total antioxidant capacity, vitamin C levels, total phenolic content, superoxide dismutase (SOD) activity, malondialdehyde (MDA) levels, and reduced glutathione (GSH) concentrations.

**Results:**

The results demonstrated significant age-dependent declines in several antioxidant parameters in control animals, including DPPH scavenging activity, total antioxidant capacity, vitamin C levels, total phenolic content, and SOD activity. Ginger supplementation produced differential effects based on both dose and age. While 3-month-old mice showed decreased DPPH radical scavenging with ginger supplementation, both 6- and 12-month-old mice exhibited significantly increased activity. Higher-dose (1.8%) ginger supplementation enhanced GSH levels across all age groups, with effects being most pronounced in older mice. SOD activity remained unaffected by ginger supplementation across all groups. MDA levels were significantly reduced by 1.8% ginger supplementation in 3-month-old mice, with smaller, dose-dependent but non-significant reductions in older groups.

**Discussion:**

These findings demonstrate that ginger’s effects on liver antioxidant systems are both dose- and age-dependent, with generally stronger beneficial effects observed at higher doses and in older animals. The observed dose- and age-dependent variations emphasize the importance of personalized supplementation strategies and provide a foundation for future research into the molecular mechanisms underlying ginger’s antioxidant effects.

## 1 Introduction

Oxidative stress occurs when there is an imbalance between the production of reactive oxygen species (ROS) and the body’s ability to detoxify these reactive intermediates or repair the resultant damage (Burton and Jauniaux, 2011; Afzal et al., 2023). The liver represents a key target that is often attacked by these reactive species (Sanchez-Valle et al., 2012). Many external factors such as drinking alcohol, overusing drugs, exposure to toxins, viruses, and smoking, and internal factors like obesity and insulin resistance can increase ROS production in the liver (Manna and Jain, 2015; Muriel and Gordillo, 2016). To counteract this oxidative damage, the liver, which is the central metabolic organ for detoxification, has a sophisticated defense system. Specifically, it produces more antioxidants under stress conditions through both enzymatic and non-enzymatic mechanisms (Caetano et al., 2013; Li et al., 2015). Enzymatic antioxidants, such as superoxide dismutase (SOD), alongside non-enzymatic antioxidants like vitamin C, among others, are important in neutralizing the adverse effects of ROS and maintaining hepatic function (Cichoz-Lach and Michalak, 2014). Aside from external stressors, age is another key factor for the redox balance of the body, with the activities of key antioxidant enzymes such as SOD tending to decline with age, which is associated with increased oxidative damage due to reduced ability to neutralize ROS (Sohal et al., 1990; Tian et al., 1998). When antioxidant defenses get overwhelmed, the imbalance caused due to oxidative stress can disrupt cellular homeostasis, leading to inflammation, fibrosis, and metabolic dysfunction (Cahill et al., 2006; Kattoor et al., 2017; Masenga et al., 2023).

Dietary supplements with antioxidant properties are often used to help manage liver diseases caused by oxidative stress (Li et al., 2015). Examples include silymarin, curcumin, fenugreek seeds, vitamin C, vitamin E, and selenium, among others (Almeida et al., 2011; Tewari et al., 2020; Ahamed et al., 2022; Mohammadi et al., 2024). These supplements work by neutralizing excessive ROS and reactive nitrogen species (RNS), which, if unchecked, can severely damage cellular lipids, proteins, and DNA. By doing so, they not only preserve cellular integrity but also support critical regulatory pathways involved in maintaining the oxidative-reductive balance (Kurutas, 2016). Among natural supplements with antioxidant properties, several prior works have demonstrated that ginger (*Zingiber officinale*) exhibits promising effects in enhancing the body’s antioxidant defenses and protecting against oxidative damage (Ballester et al., 2023; Mustafa and Chin, 2023; Ayustaningwarno et al., 2024). Recent works have also emphasized that ginger constituents possess potent antioxidant, anti-inflammatory, and anticancer properties, which are critical in regulating oxidative stress and protecting liver health, especially in age-related liver dysfunction (Bekkouch et al., 2022; Ozkur et al., 2022). The antioxidant potential of ginger is largely attributed to its major bioactive phenolic compounds, including [6]-gingerol, [8]-gingerol, [10]-gingerol, [6]-shogaol, and zingerone, which are abundant in fresh or dried rhizome extracts (Mao et al., 2019; Wu et al., 2023). These compounds have been shown to exert antioxidant activity by both direct radical scavenging (e.g., against DPPH, superoxide, hydroxyl, and nitric oxide radicals) and indirect modulation of cellular defense pathways (Peng et al., 2015). In particular, [6]-gingerol and [6]-shogaol can activate the Nrf2–ARE pathway, promoting transcription of cytoprotective genes like HO-1, NQO1, and GCLC, which help regulate glutathione metabolism and maintain redox homeostasis (Kim and Jang, 2016; Hong et al., 2020). Moreover, these compounds can inhibit NF-κB signaling, thereby reducing oxidative damage linked to chronic inflammation. These pleiotropic effects on redox-sensitive transcription factors make ginger a compelling candidate for dietary antioxidant intervention, particularly in age-associated liver dysfunction where these pathways are often dysregulated. Additionally, research has shown that ginger extracts can effectively scavenge superoxide, hydroxyl, and nitric oxide radicals in a dose-dependent manner (Jagetia et al., 2004). Such activity may support minimizing cellular damage and maintaining redox balance. In human cell studies, such as those conducted on C28/I2 chondrocytes, pretreatment with ginger extract reduced ROS levels, inhibited lipid peroxidation, and enhanced the expression of antioxidant enzymes, showing its role at a cellular level (Hosseinzadeh et al., 2017). Although ROS are deeply involved in liver injury, leading to the loss of its structure and function, the potential of ginger in counteracting such oxidative effects through various pathways is worth exploring (Banerjee et al., 2023). Building on *in vitro* findings, animal studies have further highlighted ginger’s therapeutic potential in diverse conditions associated with oxidative stress, for example, in the context of nephrotoxicity caused by agents like gentamicin and cadmium (Hegazy et al., 2016; Gabr et al., 2019) and in Polycystic Ovary Syndrome (PCOS), among other disorders (Novakovic et al., 2024). While numerous studies have shown that ginger has strong antioxidant properties, limited research has explored dose-dependent *in vivo* effects. The significance of such studies, is for example, highlighted in a work conducted on STZ-induced diabetic rats, demonstrated that ginger not only reduces oxidative stress markers such as malondialdehyde (MDA) but also reduces genetic damage caused by oxidative stress in a dose-dependent manner (Kota et al., 2012). Moreover, recent clinical research showed that ginger supplementation improved the levels of liver stress-associated biomarkers (most consistently ALT) in patients (Rahimlou et al., 2016; Rafie et al., 2020; Matin et al., 2024b). Additionally, taking in consideration the age-dependency of the regulation of liver antioxidant defense systems it is of interest to examine how ginger’s effects on liver redox balance may vary with age. While there is still a lack of conclusive research on this specific aspect, previous works have explored diverse ginger bioactivities that support putative general anti-aging action (Matin et al., 2024a).

On the background of the outlined prior research, the goal of the present study is to explore how ginger (Zingiber officinale) affects the liver’s antioxidant defense system in mice, focusing on the impact of different doses and ages. While planning this research work, we hypothesized that ginger would improve the liver’s antioxidant defenses at moderate to high doses, and that the effects will be stronger in older mice.

## 2 Materials and methods

### 2.1 Experimental setup

**FIGURE 1:**
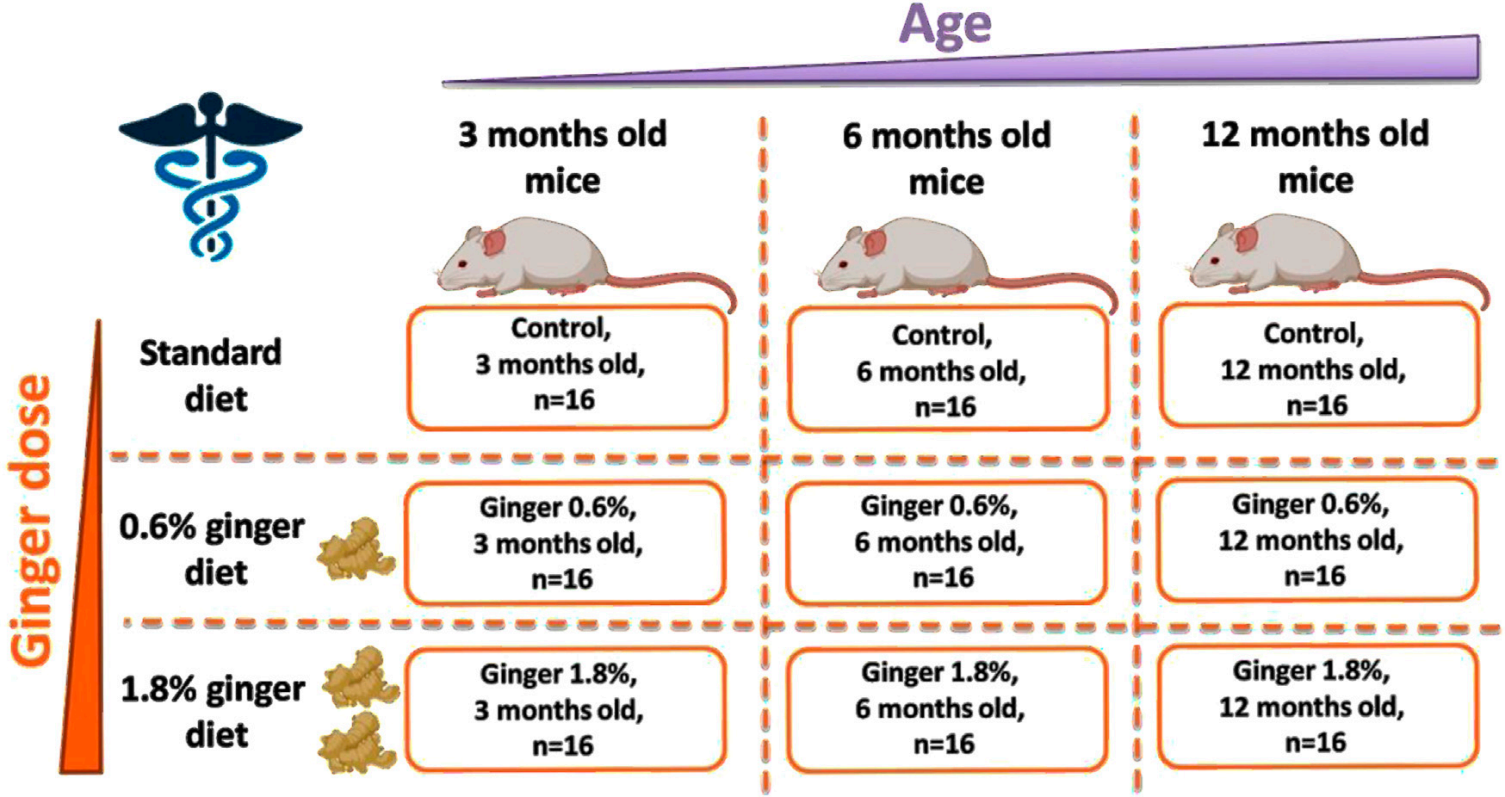
Scheme of the conducted ginger supplementation experiment.

The study was carried out with 144 Swiss Webster male mice, that were 3 months (n = 48), 6 months (n = 48), or 12 months (n = 48) old at the start of the supplementation (Figure 1). The Swiss Webster mouse strain was selected due to its widespread use in nutritional, toxicological, and pharmacological studies (Meek and Olson, 2007; Abd El-Salam and Hassan, 2017; Glavas et al., 2019). This outbred strain exhibits robust hepatic function and age-dependent redox alterations that make it suitable for modeling liver oxidative stress and assessing dietary antioxidant interventions. Furthermore, Swiss Webster mice are genetically heterogeneous, which allows for broader applicability of the findings and reduces the risk of strain-specific bias in response to dietary supplementation.

Only male Swiss Webster mice were used in this study to reduce variability associated with the estrous cycle, which can influence hormonal regulation of antioxidant enzyme systems and confound interpretation of redox-related endpoints. This approach allows for more consistent assessment of age and dose-related effects of ginger supplementation. However, it is acknowledged that sex-based differences in antioxidant capacity exist, and future studies should aim to include female mice to assess the potential for sex-specific responses to ginger supplementation.

The animals were maintained in standard cages (with four mice per cage), at the temperature of 22°C under standard conditions with 12 h of daylight and 12 h of darkness, with access to food and water. Both food and water were provided *ad libitum* throughout the entire study period, ensuring unrestricted access to standard or ginger-supplemented diets and hydration. For 3 months of supplementation period, the animals were fed with standard mouse feed (Control) or standard diet supplemented with 0.6% (Ginger 0.6%) or 1.8% (Ginger 1.8%) dried ginger (*Z. officinale*) powder (5% gingerols, supplied by Sabinsa Corporation, 20 Lake Drive, East Windsor NJ 08520, United States). Ginger powder was stored at room temperature in a foil-lined, airtight zip bag, protected from light to prevent degradation and oxidation. The ginger-supplemented fodder was prepared freshly by mixing standard feed with the respective amounts of ginger powder (0.6% or 1.8%, corresponding to 0.6 g or 1.8 g per 100 g of fodder) twice a week. After 3 months of supplementation, all animals were anesthetized with diethyl ether and decapitated. Immediately after decapitation, the liver was isolated and further processed for subjection to the described analytical procedures.

The total number of animals (144 mice) was determined based on allocating n = 16 mice per group, with three dietary treatments (control, 0.6% ginger, 1.8% ginger) across three age groups (3, 6, and 12 months). This sample size was chosen to ensure sufficient statistical power to detect medium-to-large effect sizes (Cohen’s f ≥ 0.25) using one-way and two-way ANOVA with multiple comparisons. While a formal *a priori* power analysis was not performed during the initial design phase, assuming an alpha of 0.05 and a power (1-β) of 0.80, the required sample size per group for medium effect detection using ANOVA would typically fall within the range of 12–16 animals per group (Faul et al., 2007). Future studies may refine group sizes using precise *a priori* calculations based on pilot effect sizes.

The selected ginger concentrations (0.6% and 1.8% w/w in the diet) were based on previous rodent studies that demonstrated antioxidant and hepatoprotective effects at similar levels. For instance, Ahmed et al. and Mallikarjuna et al. reported that 1% ginger in rat diets significantly improved oxidative stress biomarkers and liver function (Ahmed et al., 2008; Mallikarjuna et al., 2008). Moreover, dose-dependent antioxidant responses have been observed with 1% and 2% dietary ginger in rats (Shanmugam et al., 2011). In our study, 0.6% served as a moderate dose comparable to those previously shown to be effective, while 1.8% was selected to probe enhanced biological activity without exceeding known safe dietary limits in rodents. These levels also correspond to human-equivalent doses of approximately 3–10 g of ginger daily for a 70-kg adult, which aligns with the range used in multiple clinical supplementation studies (Anh et al., 2020).

The work was performed in the frame of animal handling permission No. 14186201 in accordance with national regulations (Act on the Protection of Animals Used for Scientific or Educational Purposes of 15 January 2015).

### 2.2 DPPH (1,1-diphenyl-2-picrylhydrazyl) radical scavenging assay

The potential of liver homogenates to scavenge the free synthetic DPPH radical was determined according to the procedure described by Brand-Williams et al. (1995). A 150 mg portion of liver tissue was cut and mixed with 1.5 mL of ultrapure methanol containing 1% acetic acid. The sample was then homogenized, and incubated in a water bath at 40°C for 2 h. Following incubation, the tubes were removed, allowed to cool briefly, and then centrifuged at 4,000 RPM for 10 min. Next, 0.5 mL of supernatant was mixed with the same volume of ethanolic solution of 0.5 mM DPPH, which was previously diluted to yield an absorbance of 0.9 when measured at a wavelength of λ = 517 nm. Next, the obtained mixture was thoroughly mixed and incubated in a dark and cool place for 30 min for stabilization of the color. Finally, Cary 50 Bio UV-VIS spectrophotometer was used for extinction measurements at a wavelength of λ = 517 nm with the use of Cary WinUV software.

### 2.3 Antioxidant capacity

The liver samples were handled according to the instructions of the Antioxidant Assay Kit by Cayman Chemical (Ann Arbor, MI, United States), according to the manufacturer’s recommendations. The liver tissue (50 mg) was homogenized in 1 mL of Assay Buffer (1X), centrifuged at 10,000 × *g* for 10 min (at 4°C), and the supernatant was promptly used for the test. According to the manufacturer’s instructions, the assay was further conducted with the use of 96-well plates in a 210 µL of total volume per well for 5 min, with the inclusion of 10 µL of Trolox standards in each handled plate. After the end of the incubation period, absorbance was read at 750 nm. A Trolox standard curve was plotted, and the antioxidant capacity of the samples was calculated and expressed as millimolar (mM) Trolox equivalents.

### 2.4 Determination of vitamin C

Vitamin C levels in livers of the experimental animals was determined with a LambdaBio-20 spectrophotometer (Perkin Elmer, Waltham, MA, United States) according to the method described by Omaye et al. (1979) with modification by Jóźwik et al. (2012). For sample preparation, 300 mg portion of liver tissue was weighed and placed into a 5 mL Eppendorf tube. A total of 1.5 mL of phosphate buffer (pH 7.0) was added to the liver tissue. The sample was homogenized thoroughly to ensure uniform consistency. For deproteination and supernatant preparation, 500 µL aliquot of the homogenized sample was transferred into a test tube. An equal volume (500 µL) of tri-chloro-acetic acid (TCA) was added, and the mixture was vortexed thoroughly. The sample was centrifuged at 3,000 RPM for 10 min. A 500 µL aliquot of the resulting supernatant was carefully collected into a clean Eppendorf tube. For colorimetric reaction development, 200 µL of phosphoric acid (H_3_PO_4_) was added to the supernatant, followed by vortexing. Next, 200 µL of 2,2-dipyridyl was added and the solution vortexed. Lastly, 100 µL of ferric chloride (FeCl_3_) was added and the solution was vortexed. Two blank samples using 500 µL of TCA were prepared for reference. The reaction mixture was incubated at 37°C for 1 h. Following incubation, the absorbance of the samples was measured at 525 nm using a spectrophotometer.

### 2.5 Determination of total phenolic content

The total phenolics content of liver homogenates was assessed following the modified method by Škerget et al. (2005) through spectrophotometric measurement of the colorimetric redox reaction upon application of the Folin-Ciocalteu reagent. Preparation of supernatant was the same as the procedure of DPPH described above. 0.5 mL of supernatant were transferred to 6 mL tubes and mixed with 2.5 mL of Folin-Ciocalteu reagent that was diluted 10-fold with demineralized water. Next, the samples were thoroughly mixed and left for 6 min, then 2 mL of saturated sodium carbonate solution was added. The following step was an incubation for 30 min at 40°C (to allow the development of a stable blue color). Again centrifugation was done for 5 min at the speed of 3000 RPM. Finally, the absorbance of the samples was measured at 765 nm wavelength in comparison to a blank sample of double-distilled H_2_O (0.5 mL). Evaluation of the results was performed with a calibration curve based on the absorbance of Gallic acid standard solutions in the range 0–0.5 mg/mL. The final results were expressed as milligrams of Gallic acid equivalents (GAE) per Gram of tissue.

### 2.6 Superoxide dismutase activity assay

The liver tissue samples were perfused with phosphate-buffered saline (PBS; pH 7.4), and homogenized in chilled HEPES buffer (20 mM, pH 7.2, containing 1 mM EDTA, 70 mM sucrose, and 210 mM mannitol). The chilled homogenates were then centrifuged for 5 min at 1,500 x *g* (4°C). The samples were then promptly tested for SOD activity with the Cayman Chemical Superoxide Dismutase Assay Kit (Ann Arbor, MI, United States) according to the manufacturer’s manual. The measurement setup included 96-well plates and a total volume of 230 µL of total volume per well, and SOD standard solutions were included on each assayed plate. The absorbance was determined at 450 nm wavelength using Synergy4 microplate reader (Biotek; Winooski, VT, United States). A SOD standard curve was plotted, and the SOD activity of the test samples was calculated and expressed in U/mL.

### 2.7 Determination of malondialdehyde levels

MDA levels in liver tissue were determined using the Thiobarbituric Acid Reactive Substances (TBARS) Assay Kit (Cayman, Ann Arbor, MI, United States) following the manufacturer’s instructions. Briefly, 25 mg of liver tissue was weighed into a 1.5 mL test tube, and 250 µL of RIPA buffer was added. The mixture was homogenized while being kept on ice. The homogenized samples were centrifuged at 1,600 × g for 10 min at 4°C, and the resulting supernatants were stored on ice. The tissue homogenates did not require dilution before the assay. For the colorimetric assay, 100 µL of each sample or standard was added to appropriately labeled microcentrifuge vials, followed by the addition of 100 µL of 10% trichloroacetic acid (TCA) assay reagent. The mixture was swirled to ensure thorough mixing before the addition of 800 µL of Color Reagent, after which the vials were vortexed. The vials were then incubated in a boiling water bath for 1 h. After incubation, the vials were transferred to an ice bath for 10 min to stop the reaction, followed by centrifugation at 1,600 × g at 4°C. From each vial, 200 µL of the supernatant was transferred (in duplicate) to a clear plate, and the absorbance was measured at 530–540 nm using a Cary Varian 50Bio spectrophotometer (Santa Clara, CA, United States). The MDA concentration was calculated using a calibration curve generated from the MDA standard provided by the manufacturer, with a concentration range of 0–50 µM.

### 2.8 Determination of reduced glutathione concentration

Reduced glutathione (GSH) concentrations were determined according to the method of Beutler et al. (1963), with minor modifications, using DTNB as the chromogenic reagent and absorbance measured at 412 nm (Beutler et al., 1963). The liver tissue sample was homogenized at a ratio of 50 mg per 250 μL of 0.1 M of phosphoric buffer containing 0.01 M of EDTA (pH = 7.4). The homogenized sample was centrifuged for 10 min at 3000 RPM. For deproteinization of the sample, a mixture was prepared by adding 100 µL of 10% TCA, 100 µL of the supernatant, and 100 µL of 10 mM EDTA and vortexed. The mixture was then left to stand for 10 min. Following this, it was centrifuged again at 3000 RPM for 10 min. For the preparation of the sample and blank solutions, 230 µL of water was added to the plates. In the sample wells, 30 µL of 3.2 M Tris-HCl (pH 8.1), 10 µL of 10 mM EDTA, 25 µL of the obtained supernatant, and 10 µL of 3 mM DTNB were added. For the blank, 30 µL of 3.2 M Tris-HCl (pH 8.1), 20 µL of 10 mM of EDTA, 10 µL of TCA and 10 µL of 3 mM DTNB were added. Plate was placed on the shaker and incubated at room temperature for 5 min. Finally, the absorbance was measured at 412 nm. Bio-Tek Synergy4 microplate reader (Winooski, VT, United States) was used to determine absorbance at 412 nm and data on reaction kinetics. The obtained results were evaluated with the Gen5 program (BioTek), and ultimately the glutathione levels were calculated as μM concentration values.

### 2.9 Statistical analysis

The data are presented as mean ± standard deviation (SD). Data analysis was performed using the Real Statistics Resource Pack software (Release 8.9.1; copyright 2013–2023; Charles Zaiontz; www.real-statistics.com). Analysis of Variance (ANOVA) at *α* = 0.05, with Bonferroni correction for multiple comparisons, was used to determine statistical significance. Tukey’s Honest Significant Difference (HSD) test was conducted as a follow-up to ANOVA. In the generated graphs, statistically significant differences (p ≤ 0.05, p ≤ 0.01, and p ≤ 0.001) were indicated by one, two, or three asterisks, respectively.

## 3 Results

The effects of dietary ginger supplementation on liver antioxidant defense systems were evaluated across different age groups of mice through multiple biochemical and enzymatic tests.

**FIGURE 2:**
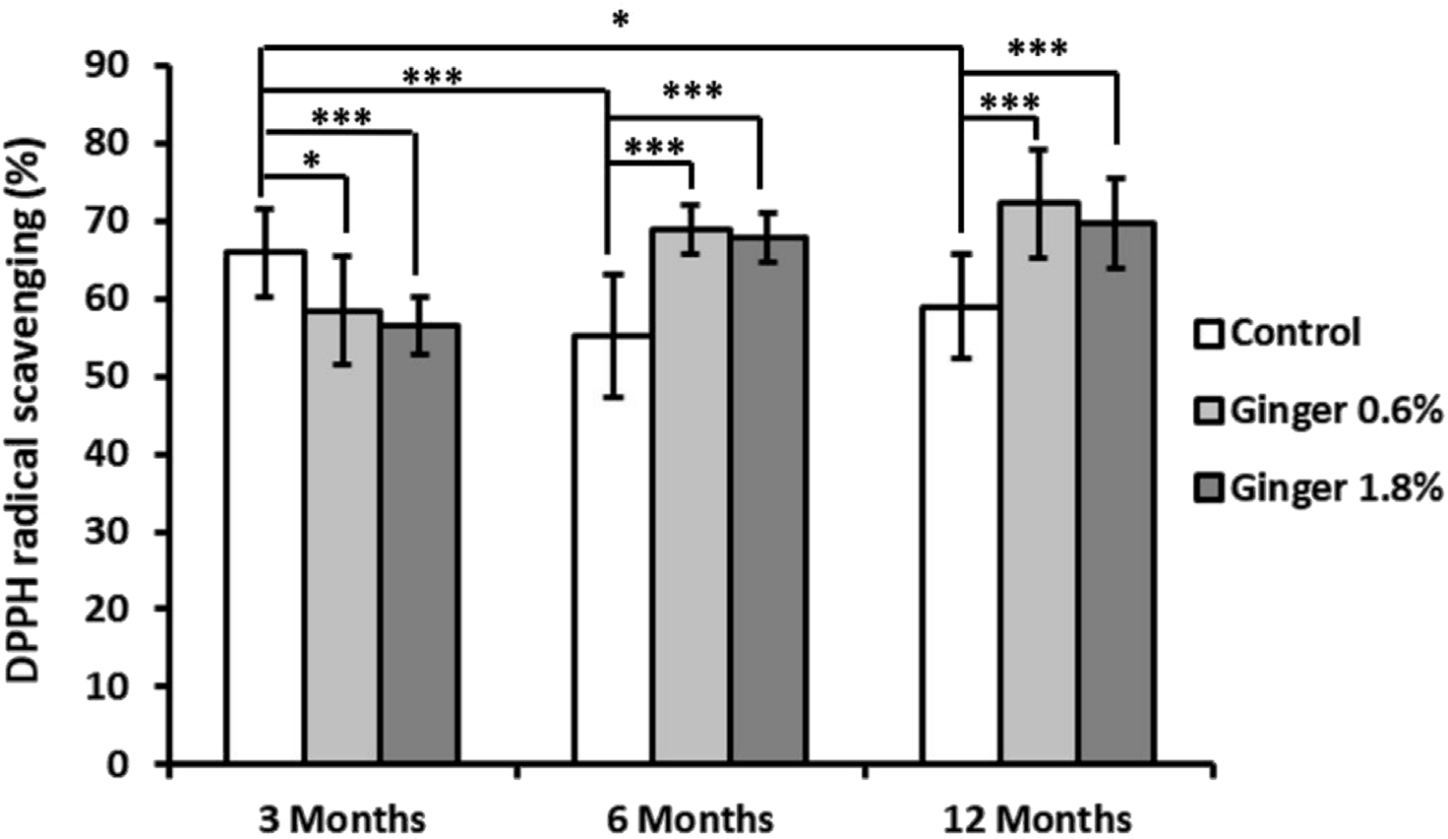
Liver DPPH radical scavenging activity. Data are presented as the percentage of DPPH radical scavenging across different age groups (3, 6, and 12 months) and dietary treatments (control, 0.6% ginger, 1.8% ginger). Values are shown as mean ± standard deviation (n = 16). Statistical significance between groups is indicated (ANOVA/Tukey HSD; *p ≤ 0.05; ***p ≤ 0.001).

DPPH radical scavenging activity in liver tissue demonstrated both age- and dose-dependent responses to ginger supplementation (Figure 2). In the control group animals, there was a decrease in the DPPH scavenging activity with advancement of age (65.88% ± 5.69%, 55.19% ± 7.87%, and 59.00% ± 6.82%, for the 3-month-old, 6-month-old, and the 12-month-old animals, respectively; with a significance of p ≤ 0.001 in the 6-month-old control group animals and p ≤ 0.05 in the 12-month-old control animals, both in comparison to the 3-month-old control group). In the 3-month-old mice, both 0.6% and 1.8% ginger supplementation significantly decreased DPPH radical scavenging activity compared to control (65.88% ± 5.69% for the control, 58.46% ± 6.91% for the 0.6% ginger supplemented animals, and 56.52% ± 3.70% for the 1.8% ginger supplemented animals). This effect was more pronounced in the 1.8% ginger supplemented group (p ≤ 0.001). The 6-month-old mice and the 12-month-old mice groups showed an inverse pattern, with significant increase (p ≤ 0.001) in the DPPH radical scavenging in response to ginger supplementation, thus exhibiting ginger supplementation-induced reversal of the age-dependent decrease observed for the control animal groups.

**FIGURE 3:**
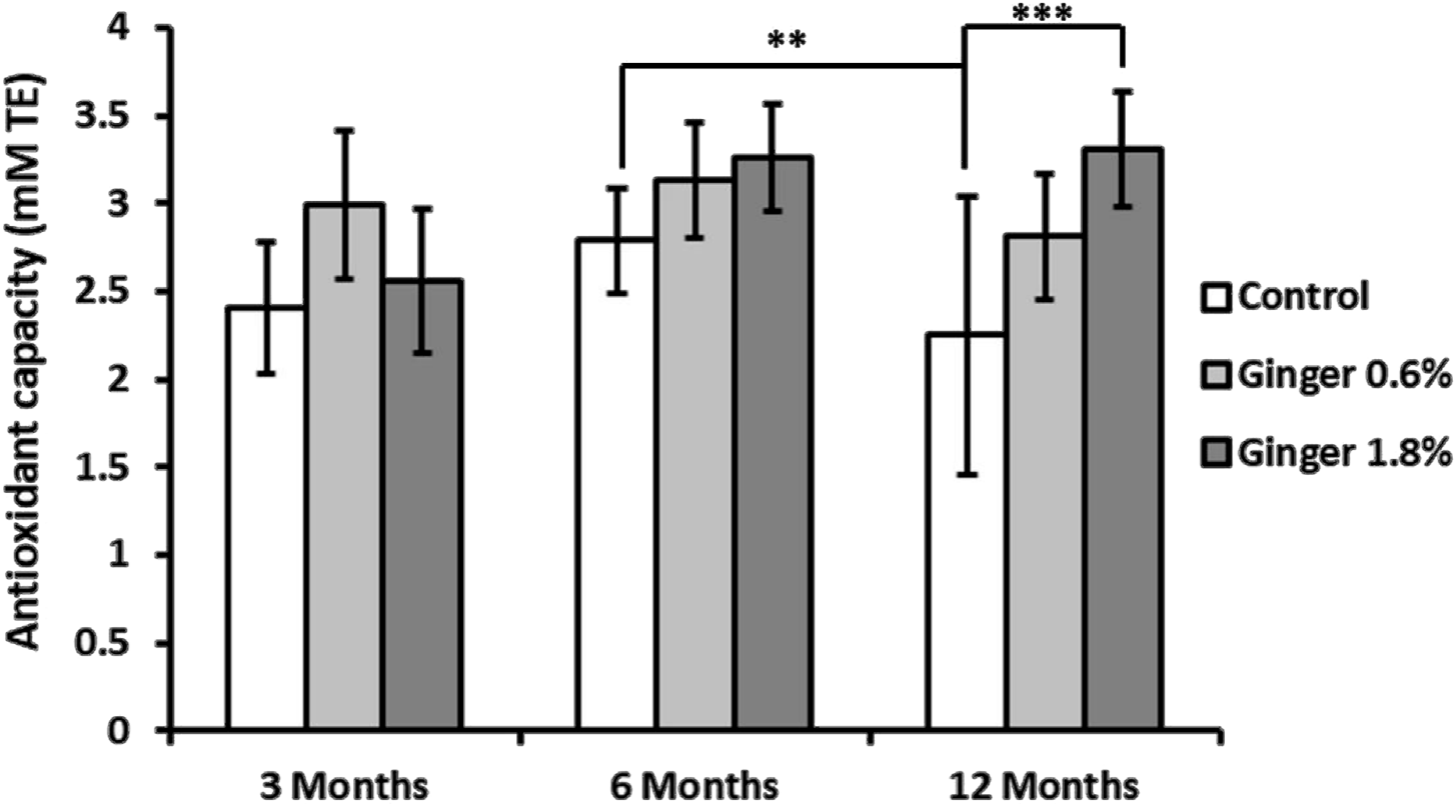
Liver total antioxidant capacity. Total antioxidant capacity of liver homogenates was determined as described in detail in the Methods section. The results are expressed in millimolar (mM) Trolox equivalents (TE). Data represent mean ± standard deviation (n = 8), with significance denoted by asterisks. (ANOVA/Tukey HSD; **p ≤ 0.01; ***p ≤ 0.001).

Total antioxidant capacity, measured as Trolox equivalents, declined in the control animals (2.40 ± 0.40, 2.79 ± 0.30, and 1.86 ± 0.66 Trolox equivalents for the 3-month-old, 6-month-old, and the 12-month-old animals, respectively) with significant decrease especially in the 12-month-old control group (p ≤ 0.01, Figure 3). Ginger supplementation overall displayed a pattern of enhanced total antioxidant capacity across all age groups, with the effect being most pronounced in the 1.8% supplemented 12-month-old group (1.86 ± 0.66 Trolox equivalents in the control group versus 3.31 ± 0.33 Trolox equivalents in the supplemented animals; with p ≤ 0.001, in comparison to the 12-month-old control group).

**FIGURE 4:**
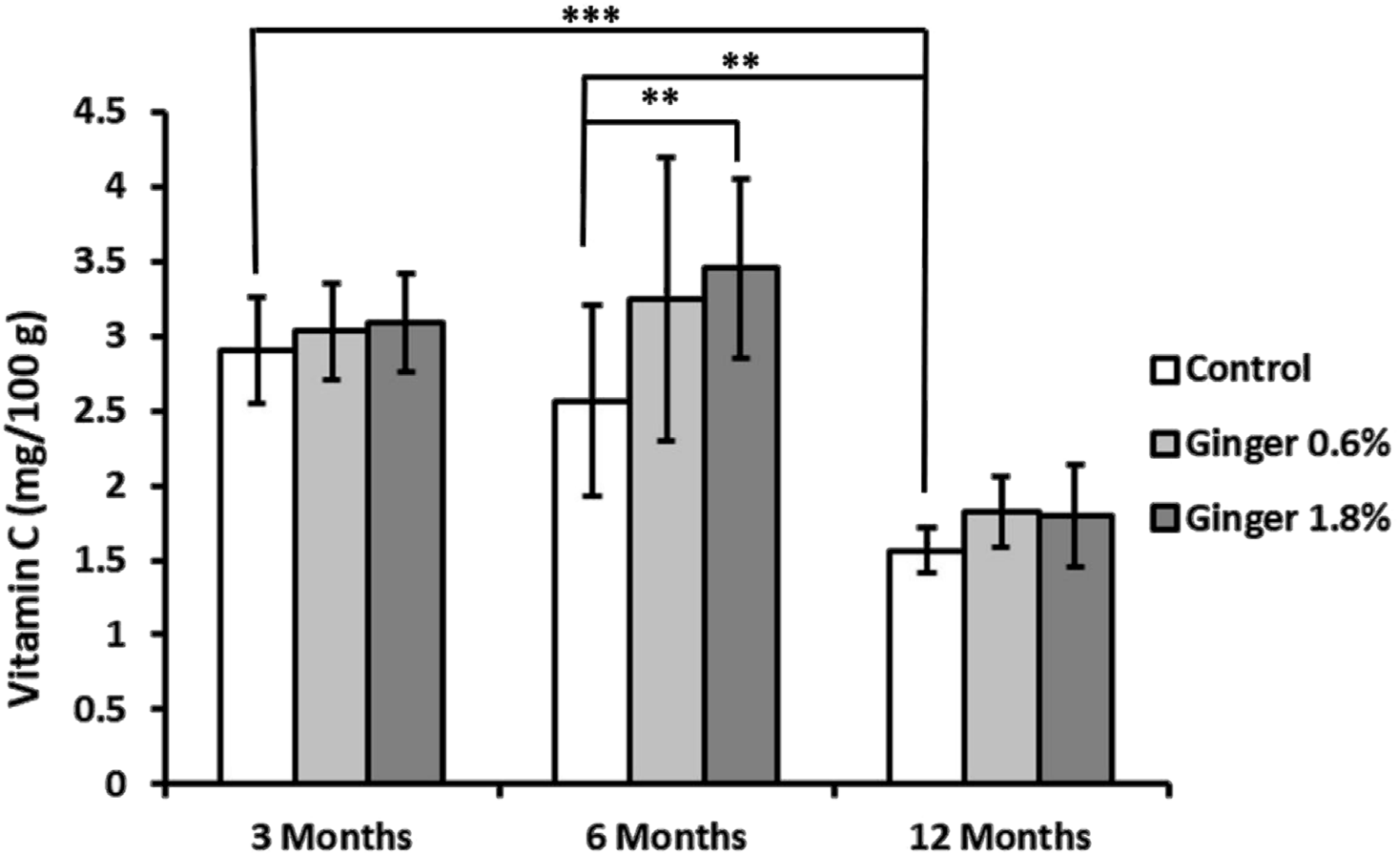
Liver vitamin C levels. Vitamin C concentrations in liver tissue were determined spectrophotometrically as described in the Methods section. The results are expressed as milligrams of vitamin C per Gram of tissue. Data represent mean ± standard deviation (n = 8). Statistical significance between groups is indicated (ANOVA/Tukey HSD; **p ≤ 0.01; ***p ≤ 0.001).

Liver vitamin C levels showed an age-dependent decline in control animals (2.91 mg ± 0.36 mg, 2.56 mg ± 0.64 mg, and 1.56 mg ± 0.15 mg, for the 3-month-old, 6-month-old, and the 12-month-old animals, respectively; Figure 4), with the 12-month-old control group exhibiting approximately twice lower vitamin c levels compared to the 3-month-old control group (1.56 ± 0.15 versus 2.91 ± 0.36 mg/100 g; mean ± SD; p ≤ 0.001). Ginger supplementation overall displayed a pattern of increased vitamin C levels in comparison to the respective controls in all age groups, with a significant effect in the 6-month-old group supplemented with 1.8% ginger (2.56 mg ± 0.64 mg versus 3.46 mg ± 0.60 mg; p ≤ 0.01 compared to respective age-matched control).

**FIGURE 5:**
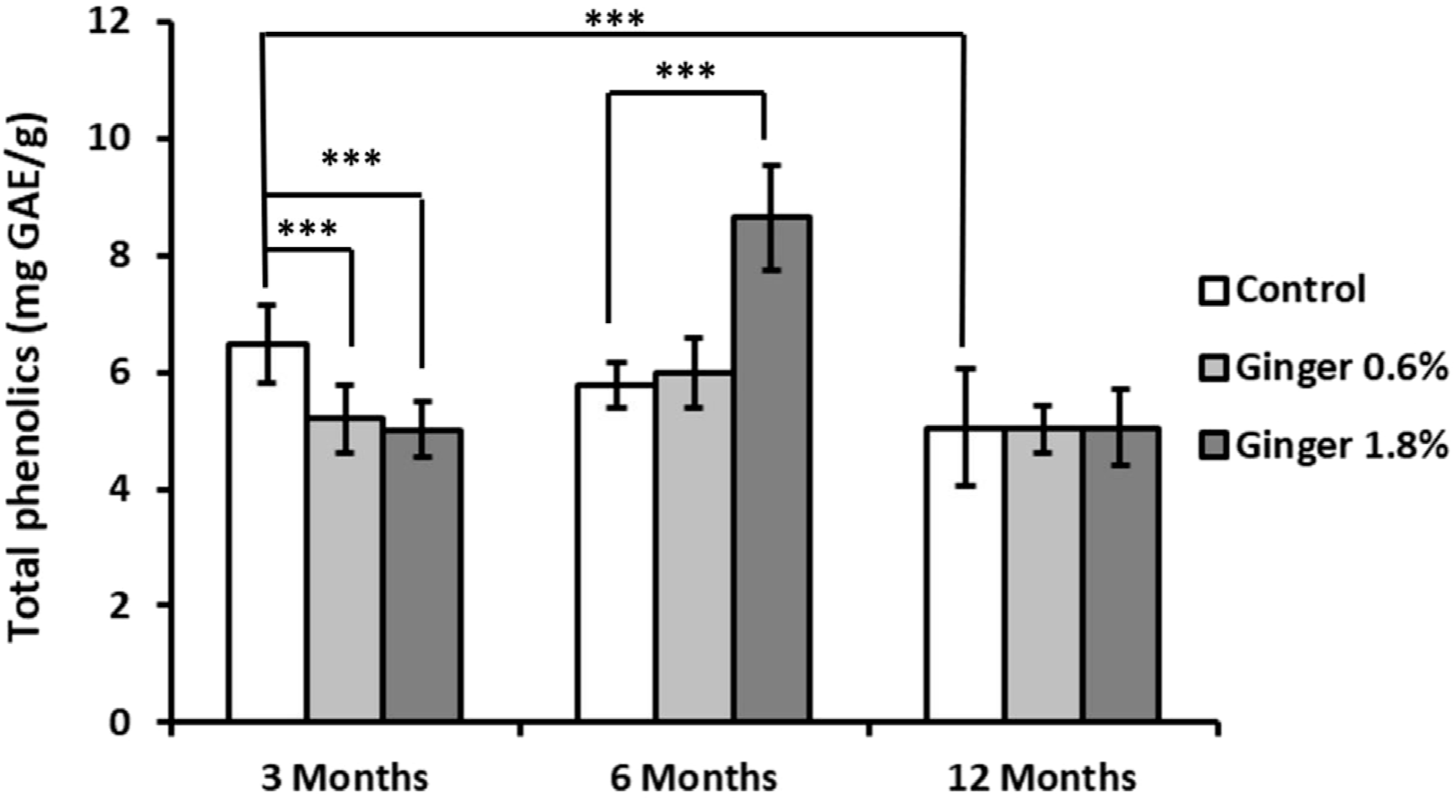
Liver total phenolic content. The total phenolic content in liver homogenates was assessed using the Folin-Ciocalteu reagent, as detailed in the Methods section. Results are expressed as milligrams of gallic acid equivalents (GAE) per Gram of tissue. Data are shown as mean ± standard deviation (n = 16). Statistical differences between groups are indicated (ANOVA/Tukey HSD; ***p ≤ 0.001).

Total phenolic content showed a tendency of age-related decline in control animals (6.47 ± 0.67, 5.77 ± 0.38, and 5.04 ± 1.00 mg GAE/g for the 3-month-old, 6-month-old, and the 12-month-old animals, respectively; Figure 5), with the 12-month-old control group showing significantly lower levels compared to the 3-month-old control group (5.04 ± 1.00 versus 6.47 ± 0.67 mg GAE/g tissue; mean ± SD; p ≤ 0.001). Ginger supplementation led to differential effects in the different age groups, with decrease of phenolic content levels in the 3-month-old animals, increase in the 6-month-old animals (especially the 1.8% supplemented group, 5.77 ± 0.38 versus 8.65 ± 0.89 mg GAE/g tissue; p ≤ 0.001 in comparison to the age-matched control), and no effect in the 12-months-old animal groups.

**FIGURE 6:**
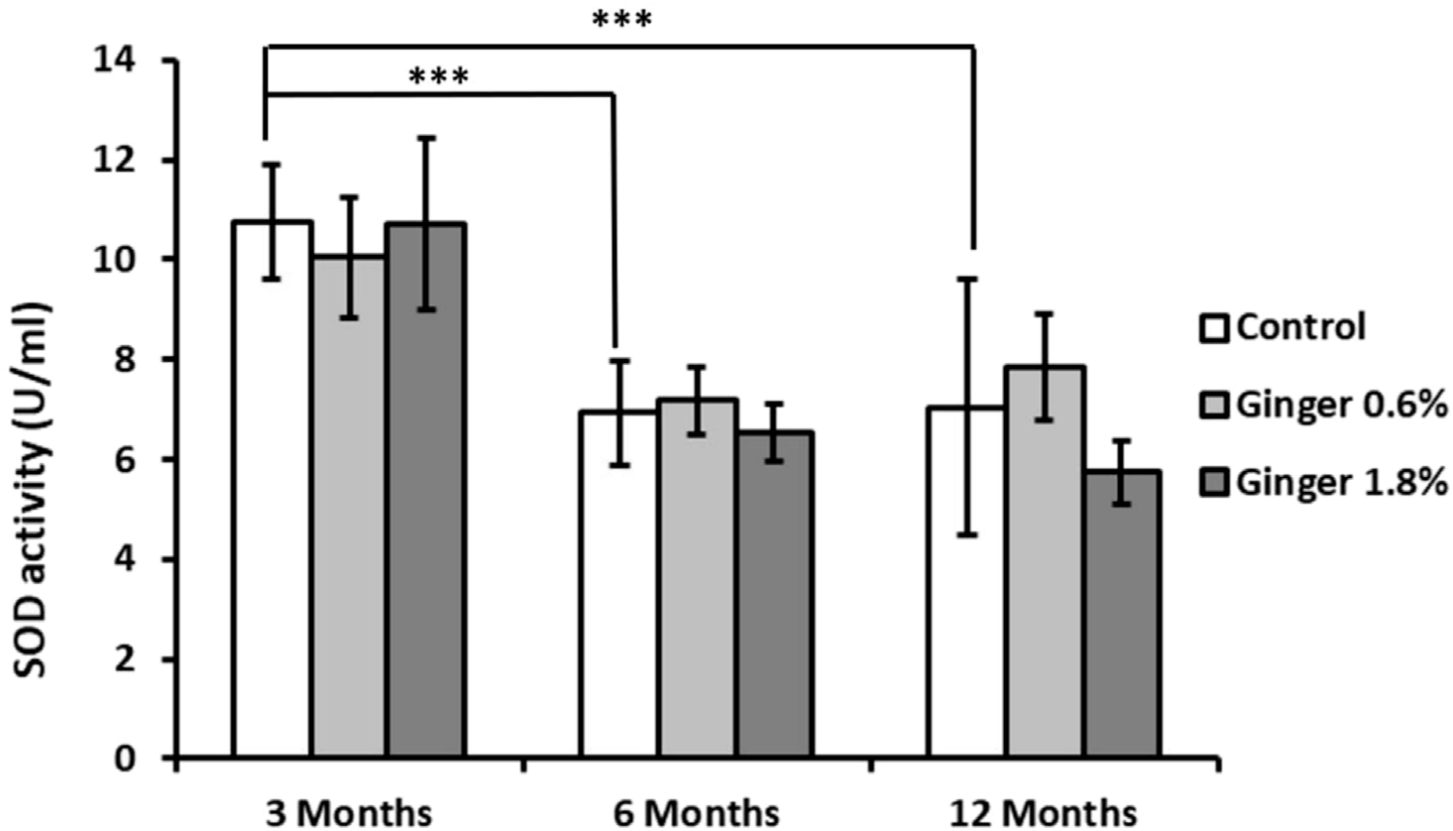
Liver superoxide dismutase (SOD) activity. Superoxide dismutase activity in liver homogenates was measured as described in the Methods section and is expressed in U/mL. Data are presented as mean ± standard deviation (n = 8). Statistical significance is indicated (ANOVA/Tukey HSD; ***p ≤ 0.001).

Superoxide dismutase (SOD) activity showed significant age-related decline in control animals (10.76 ± 1.15, 6.93 ± 1.05. and 7.04 ± 2.58 U/mL for the 3-month-old, 6-month-old, and the 12-month-old animals, respectively; Figure 6), with both the 6-month-old and the 12-month-old control group exhibiting markedly lower activity compared to the 3-month-old control group (6.93 ± 1.05 and 7.04 ± 2.58 versus 10.76 ± 1.15 U/mL; mean ± SD; p ≤ 0.001). Ginger supplementation did not produce significantly different effects on SOD activity in comparison to the respective age-matched control groups.

**FIGURE 7:**
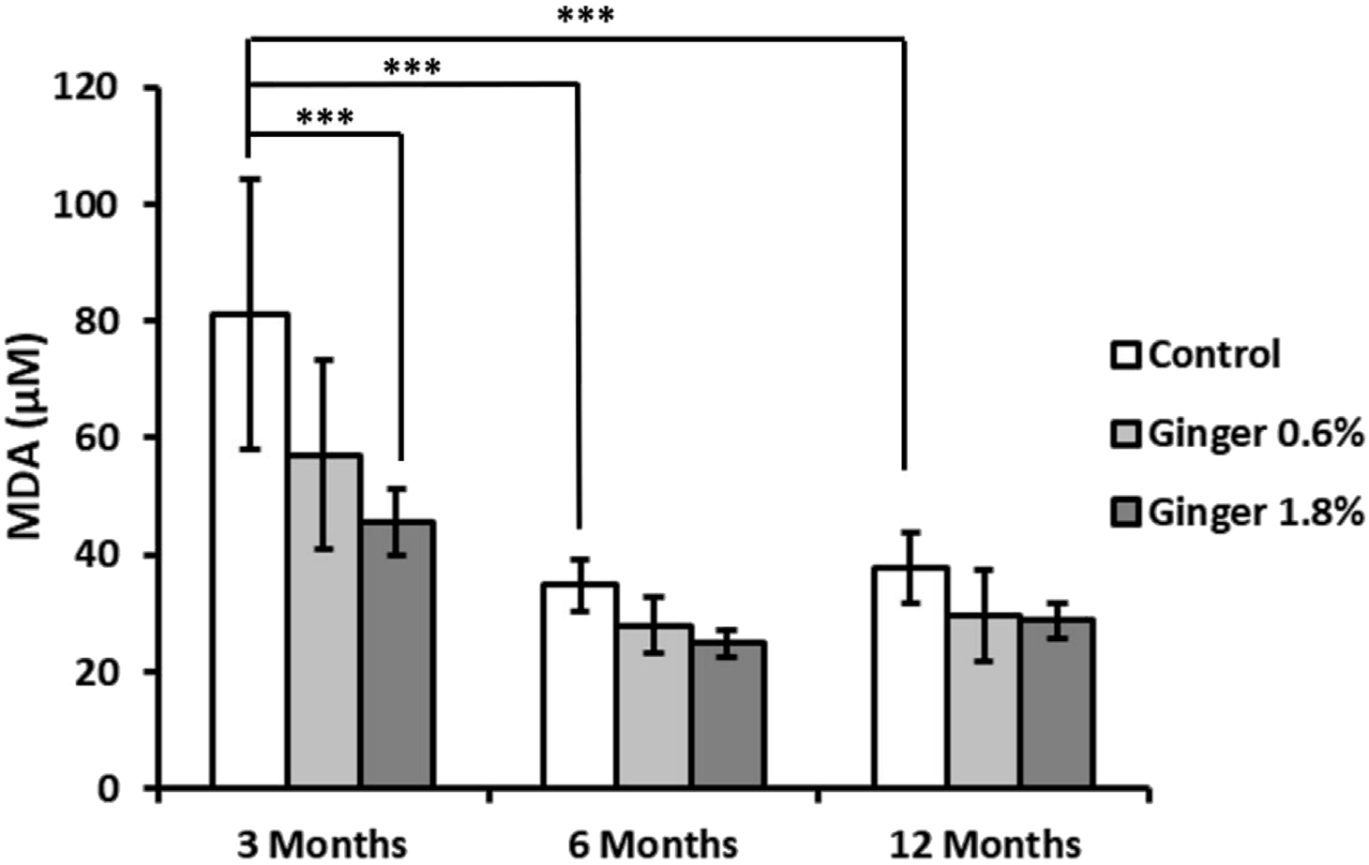
Liver malondialdehyde (MDA) levels. Malondialdehyde levels, an indicator of lipid peroxidation, were measured in liver tissue homogenates. Data are expressed as micromolar (μM) concentrations. Results are shown as mean ± standard deviation (n = 8). Statistical significance between groups is indicated (ANOVA/Tukey HSD; ***p ≤ 0.001).

Malondialdehyde (MDA) levels demonstrated a significant age-related decrease in control animals (81.08 ± 23.16, 34.86 ± 4.40, and 37.77 ± 5.89 µM for the 3-month-old, 6-month-old, and the 12-month-old animals, respectively; Figure 7), with both the 6-month-old and 12-month-old control groups exhibiting substantially lower levels compared to the 3-month-old control group (34.86 ± 4.40 and 37.77 ± 5.89 versus 81.08 ± 23.16 μM; mean ± SD; p ≤ 0.001). In 6-month-old and 12-month-old animals, ginger supplementation at both concentrations resulted in moderate decreases in MDA levels compared to age-matched controls, which did not reach statistical significance. The most pronounced effect was observed in 3-month-old animals supplemented with 1.8% ginger, where MDA levels were significantly (p ≤ 0.001; 81.08 ± 23.16 versus 45.44 ± 5.69 µM) reduced compared to the age-matched control group.

**FIGURE 8:**
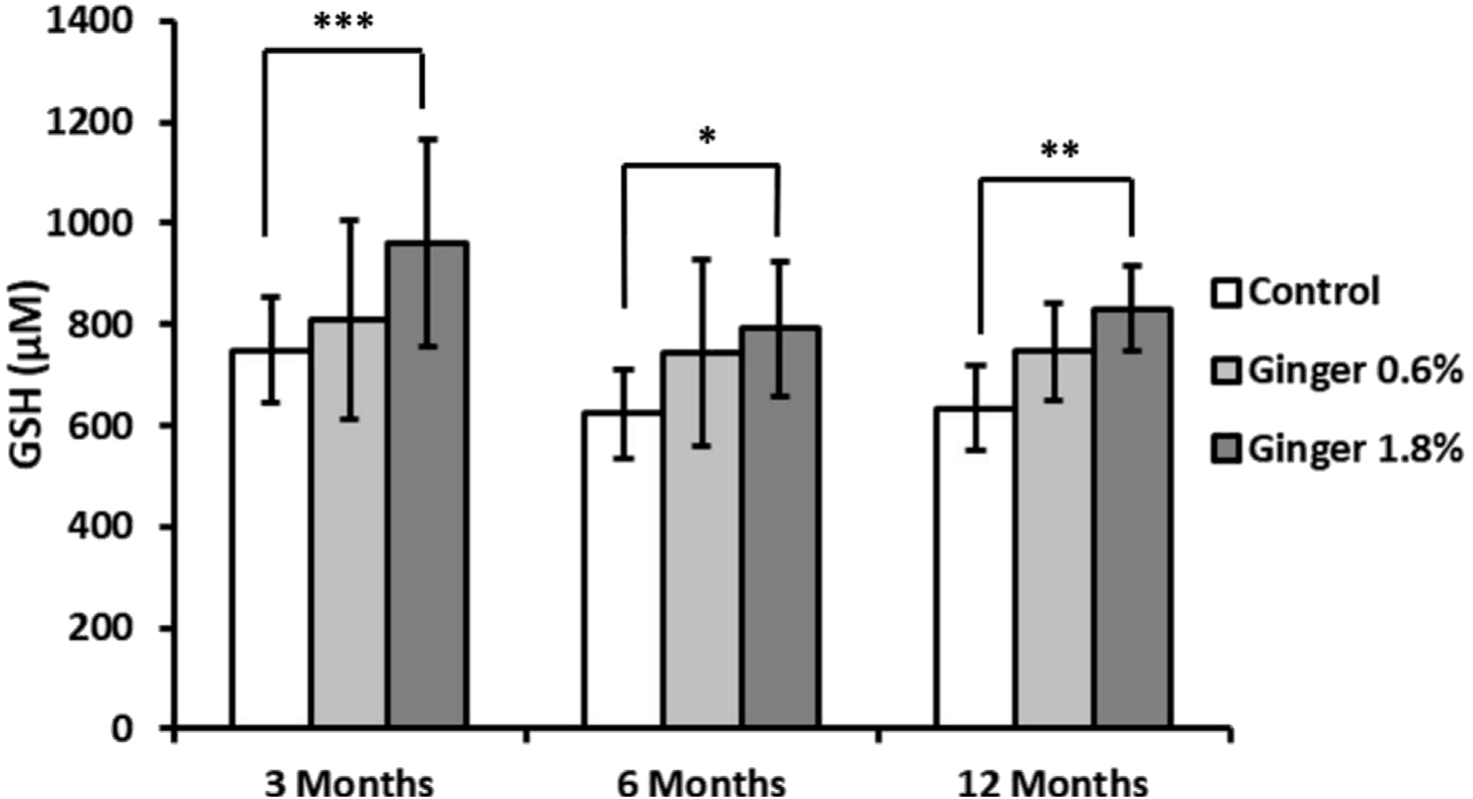
Liver reduced glutathione (GSH) levels. Reduced glutathione concentrations in liver homogenates were determined as described in the Methods section and expressed in micromolar (μM) concentrations. Data are shown as mean ± standard deviation (n = 16). Statistical significance is indicated (ANOVA/Tukey HSD; *p ≤ 0.05; **p ≤ 0.01; ***p ≤ 0.001).

Reduced glutathione (GSH) levels did not display statistically significant age-related variations in control groups animals (Figure 8). Ginger supplementation produced enhancing effects in comparison to the age-matched controls in all age groups, with the 1.8% concentration being more effective (inducing statistically significant effects with p ≤ 0.001, p ≤ 0.05, and p ≤ 0.01 in the 3-month-old, 6-month-old, and 12-month-old animal groups, with 748.07 ± 104.25 versus 962.02 ± 204.59, 623.94 ± 88.30 versus 791.75 ± 132.33, and 634.57 ± 84.85 versus 831.43 ± 83.73 μM, respectively).

## 4 Discussion

This study provides novel insights into how ginger dietary supplementation affects liver antioxidant defense systems across different age groups in mice. The results demonstrate complex age- and dose-dependent effects of ginger supplementation on various markers of oxidative stress and antioxidant capacity in liver tissue.

Our results confirm the age-dependent decline in several key antioxidant parameters, including DPPH radical scavenging activity, total antioxidant capacity, vitamin C levels, total phenolic content, and SOD activity. This aligns with previous research suggesting that aging is associated with diminished antioxidant defenses and increased oxidative stress, which contribute to liver dysfunction (Shih and Yen, 2007; Castro Mdel et al., 2012; Lozada-Delgado et al., 2020). Our findings support this paradigm and extend it by demonstrating the differential response of antioxidant defenses to ginger supplementation at various life stages. Moreover, the observed decline in parameters such as SOD activity and vitamin C levels in the control animal groups emphasizes the importance of age-specific interventions to counteract these deficits.

The dose-dependent effects of ginger supplementation were evident across most of the studied biochemical markers. At higher doses (1.8%) and most consistently in older mice, ginger supplementation increased DPPH radical scavenging activity, enhanced total antioxidant capacity, as evidenced by increased Trolox equivalents, and increased reduced GSH levels. These findings align with prior studies suggesting that ginger’s antioxidant properties are mediated through effects such as scavenging of reactive oxygen species and enhancement of endogenous antioxidant systems (Stoilova et al., 2007; Lee et al., 2011; Sueishi et al., 2019).

From a mechanistic standpoint, the antioxidant effects of ginger are likely mediated by both direct and indirect pathways. Ginger’s bioactive constituents—particularly gingerols, shogaols, paradols, and zingerone—are known to exert free radical scavenging activity, lipid peroxidation inhibition, and upregulation of endogenous antioxidant defenses. These actions are partially attributed to modulation of the Nrf2 (nuclear factor erythroid 2–related factor 2) signaling pathway, which regulates the expression of antioxidant response element (ARE)-dependent genes such as those encoding glutathione S-transferase, heme oxygenase-1 (HO-1), and NAD(P)H:quinone oxidoreductase 1 (NQO1) (Peng et al., 2015). Furthermore, ginger has been shown to inhibit inflammatory signaling cascades like NF-κB, which are often intertwined with oxidative stress responses. In aged organisms, these pathways may become dysregulated or exhibit reduced responsiveness. This could explain the enhanced efficacy of ginger observed in older animals in the present study—where the supplementation may help restore diminished redox regulation by reactivating Nrf2 signaling or replenishing depleted non-enzymatic antioxidants such as GSH and vitamin C. Moreover, pharmacokinetic alterations with age—such as slower metabolism and longer tissue retention of ginger compounds—may enhance their local effectiveness in older livers.

Although the total antioxidant capacity (TAC) appeared numerically higher in the 6-month-old control group than in the 3-month-old control group, and lower in the 1.8% ginger-supplemented group at 3 months compared to the corresponding control, these differences did not reach statistical significance. Therefore, these observations should be interpreted with caution. However, minor fluctuations in TAC between age groups could reflect transitional physiological states in antioxidant regulation during early adulthood. Some studies suggest that compensatory upregulation of endogenous antioxidant systems may transiently occur during midlife before the onset of age-related decline (Benzi et al., 1989; Shih and Yen, 2007), potentially contributing to slightly elevated TAC at 6 months. Regarding the numerically lower TAC in the high-dose ginger group at 3 months, it is possible that high-dose antioxidant supplementation in physiologically young and redox-balanced animals may trigger negative feedback regulation or subtle pro-oxidant shifts, as previously proposed in models of hormesis and antioxidant overcompensation (Calabrese and Baldwin, 2003). Although these effects were not statistically significant in our study, they highlight the importance of considering age and baseline redox status when evaluating the impact of dietary antioxidant interventions. Future studies with larger sample sizes or targeted molecular profiling could help determine whether these trends are biologically meaningful.

A key finding was the age-dependent pattern observed in DPPH radical scavenging activity. While younger (3-month-old) mice showed decreased DPPH radical scavenging with ginger supplementation, both middle-aged (6-month-old) and older (12-month-old) mice exhibited significantly increased activity. This differential response suggests that the impact of ginger on free radical scavenging mechanisms varies with age, potentially due to age-related changes in baseline antioxidant capacity as well as age-related changes of in the rate of metabolizing exogenously supplied bioactive compounds including ginger phytochemicals with antioxidant action (Kim et al., 2002; Lee et al., 2008). Meanwhile, the enhanced DPPH radical scavenging observed in older mice aligns with previous research showing ginger’s ability to combat oxidative stress that is increased with aging (Peng et al., 2015; Hosseinzadeh et al., 2017; Al Hroob et al., 2018).

The observed decrease in DPPH radical scavenging activity in 3-month-old mice supplemented with ginger, particularly at the 1.8% dose, may be explained by several interconnected mechanisms. First, young mice generally possess robust endogenous antioxidant systems, with high baseline levels of enzymatic and non-enzymatic antioxidants. Introducing additional exogenous antioxidants like ginger may lead to transient pro-oxidant effects or feedback inhibition of endogenous antioxidant responses—a phenomenon often observed in hormetic responses to phytochemicals. This biphasic behavior is supported by the concept that mild oxidative stress can activate protective mechanisms, whereas excessive antioxidant input in already-balanced systems may dampen radical signaling or paradoxically impair redox balance (Calabrese and Baldwin, 2003). Additionally, certain phenolic compounds in ginger may exhibit dual roles—acting as antioxidants at low or moderate ROS levels but exhibiting pro-oxidant effects under high-reducing or low-ROS cellular states (Maliar et al., 2023). This could explain the reduced DPPH scavenging in young mice where oxidative burden is minimal. Moreover, the higher metabolic activity and detoxification efficiency in younger animals may lead to faster metabolism and clearance of ginger phytochemicals, limiting their local accumulation and effect in liver tissue. Collectively, these age-dependent pharmacodynamic and redox-regulatory differences help explain the attenuated response in younger animals and underscore the importance of physiological context in interpreting antioxidant interventions.

The age-related differences in antioxidant response to ginger supplementation observed in this study highlight a complex interplay between baseline redox status and metabolic responsiveness. Younger mice (3 months old) displayed higher endogenous antioxidant levels in control groups, which may have limited the observable benefits of supplementation or even led to paradoxical effects (e.g., reduced DPPH scavenging and phenolic content). This may be due to a “ceiling effect,” where antioxidant systems are already well-regulated and additional stimulation is unnecessary or counterproductive. In contrast, middle-aged and older mice (6 and 12 months) exhibited significant age-associated declines in DPPH scavenging, vitamin C levels, and SOD activity—creating a physiological state of redox vulnerability. Ginger supplementation in these groups likely restored redox homeostasis by bolstering antioxidant reserves, particularly through the replenishment of GSH and enhancement of total antioxidant capacity. This aligns with prior findings that antioxidant interventions have greater benefit in aged or oxidatively stressed organisms compared to healthy young counterparts (Shih and Yen, 2007; Castro Mdel et al., 2012). These observations support the concept of age-personalized antioxidant supplementation, where physiological context determines the efficacy of dietary interventions.

The significant reduction MDA levels, particularly in younger mice receiving high-dose ginger supplementation, reflects the previously reported ability of dietary ginger to counter lipid peroxidation (Ahmed et al., 2000; Ahmed et al., 2008). However, the less pronounced effects in older animals might indicate an age-related resistance to modulation of lipid peroxidation pathways. These findings warrant further investigation into the molecular mechanisms underpinning these differences.

Interestingly, MDA levels in control animals were significantly higher at 3 months compared to 6 and 12 months, which contrasts with the typical expectation of age-related increases in lipid peroxidation. However, several factors may explain this finding. In early adulthood, mice exhibit higher metabolic rates, elevated mitochondrial respiration, and increased membrane lipid turnover, all of which can transiently elevate ROS production and enhance susceptibility to lipid peroxidation (Sohal et al., 1990). Moreover, older tissues may shift toward protein and DNA oxidation markers, with relatively less lipid peroxidation occurring at baseline (Cakatay et al., 2003; Ma et al., 2013). Therefore, MDA should not be viewed in isolation as a direct surrogate of total oxidative burden across ages. These findings highlight that age-related shifts in oxidative stress profiles can vary depending on the molecular target (lipid, protein, DNA), metabolic context, and tissue-specific vulnerability.

Superoxide dismutase activity demonstrated significant age-related decline, confirming previous findings about the deterioration of liver enzymatic antioxidant defenses with age (Reiss and Gershon, 1976; Santa Maria et al., 1996). The lack of significant changes with ginger supplementation suggests that ginger’s antioxidant effects might primarily operate through non-enzymatic pathways, rather than by modulating SOD activity directly.

The lack of a measurable effect of ginger supplementation on total SOD activity, despite improvements in other antioxidant parameters, may stem from several factors. First, the assay used in this study measured total SOD activity, without discriminating between isoforms such as SOD1 (cytosolic) and SOD2 (mitochondrial). Prior studies have shown that ginger may preferentially affect mitochondrial oxidative stress pathways and upregulate SOD2 (Homa and Wentz-Hunter, 2018), changes that could remain undetected in whole-tissue homogenates where SOD1 predominates. Second, it is possible that the antioxidant benefits of ginger in this model were mediated more through non-enzymatic antioxidants (e.g., GSH, vitamin C) or via modulation of other enzymatic systems (e.g., catalase, glutathione peroxidase), thereby limiting the need for enhanced SOD activity. Third, redox homeostasis involves complex compensatory networks. Increased glutathione or phenolic content may reduce superoxide burden, alleviating the need for further SOD induction. Finally, assay sensitivity and technical variability may also obscure subtle isoform-specific changes. Further studies examining specific SOD isoforms at the transcriptional and protein level, or subcellular localization of antioxidant effects, could help clarify these findings.

Although ginger supplementation did not produce statistically significant changes in SOD activity in any group, it is worth noting that SOD activity values in the 1.8% ginger groups at 6 and 12 months were numerically lower than those in the 0.6% ginger groups. This observation, while not statistically significant, may reflect a dose-dependent regulatory ceiling or feedback suppression of enzymatic antioxidants when exogenous antioxidant input is high. At elevated doses, ginger-derived phenolics may strongly increase non-enzymatic antioxidant reserves—such as GSH and vitamin C—which could reduce the cellular demand for SOD activity, leading to compensatory downregulation. This type of adaptive response has been reported in other antioxidant supplementation studies, where excessive exogenous antioxidant availability dampened the expression of genes of endogenous antioxidant defense proteins (Halliwell, 2000; Calabrese and Baldwin, 2003). Another possibility is that higher doses of ginger exert differential effects on antioxidant enzyme regulation depending on cellular redox state and age, which may not follow a linear dose–response pattern. However, due to the lack of statistical significance, these interpretations remain speculative and should be explored in future studies involving isoform-specific enzyme measurements and redox-sensitive gene expression profiling.

Glutathione levels showed a consistent positive response to ginger supplementation across all age groups, with the higher dose (1.8%) being more effective. This enhancement of GSH levels is particularly noteworthy given glutathione’s central role in cellular redox regulation and antioxidant defense (Zhang and Forman, 2012). The ability of ginger to boost GSH levels across different age groups suggests it could have broad applications in supporting liver antioxidant defenses.

The findings of the present study collectively support our initial hypothesis regarding ginger’s beneficial effects on liver antioxidant systems, particularly at higher doses. However, the age-dependent variations in response highlight the complexity of antioxidant system regulation and suggest that ginger’s effects may be modulated by age-related factors. The stronger responses observed in older mice for several parameters (particularly DPPH radical scavenging and total antioxidant capacity) align with our hypothesis about enhanced effects in older animals.

The results also provide insights into potential mechanisms of ginger’s antioxidant effects. The simultaneous enhancement of non-enzymatic antioxidants (vitamin C, GSH) and total antioxidant capacity, coupled with reduced lipid peroxidation (MDA), suggests multiple pathways of action. This multi-target effect is consistent with previous studies showing ginger’s diverse bioactive properties (Mao et al., 2019; Wu et al., 2023).

These findings have several potential implications for therapeutic applications. The age-dependent effects observed suggest that ginger supplementation might be particularly beneficial in supporting liver antioxidant defenses in older individuals, where natural antioxidant systems may be compromised. This aligns with prior research showing ginger’s potential versatile benefits in supporting liver health (Gao et al., 2012; AuthorAnonymous et al., 2017; Hamza et al., 2021; Guo et al., 2022). The study outcomes also hint to potential broader anti-aging benefits of ginger supplementation, in line with its previously characterized versatile bioactivity profile simultaneously contracting different hallmarks of aging (Ozkur et al., 2022; Matin et al., 2024a). Additionally, the dose-dependent effects observed in the present work support the importance of appropriate dosing in ginger supplementation strategies.

Future research could focus on elucidating the molecular mechanisms underlying the age-specific effects observed, particularly the differential responses in young versus older animals. Investigation of changes in antioxidant gene expression and protein levels could provide additional insights into how ginger modulates these systems. Additionally, longer-term studies could help understand the sustainability of these effects and potential adaptations over time.

## 5 Limitations

This study has several limitations that should be considered when interpreting the findings. While we evaluated total superoxide dismutase (SOD) activity, the assay did not distinguish between SOD isoforms (e.g., SOD1 and SOD2), nor did it assess other antioxidant enzymes such as catalase or glutathione peroxidase. Thus, potential isoform-specific or compensatory effects may have been masked in total activity measurements.

A limitation of this study is the exclusive use of male animals, which, while controlling for hormonal variability, does not account for sex-specific differences in antioxidant defense systems. Estrogen, for instance, has been shown to modulate redox signaling and influence expression of key antioxidant enzymes such as SOD and GPx. Therefore, future research should include both sexes to determine whether the antioxidant effects of ginger exhibit sex-specific patterns, particularly in the context of aging.

Further, although we observed several trends across age and dose groups, some differences—particularly those in DPPH scavenging, total antioxidant capacity, and SOD activity—did not reach statistical significance. These non-significant patterns should be interpreted with caution and may benefit from further validation with larger sample sizes or targeted molecular analyses. In addition, we did not perform transcriptomic or proteomic analyses to directly assess the molecular pathways modulated by ginger, such as Nrf2 or NF-κB signaling, which are known to regulate antioxidant defenses. These mechanistic insights remain speculative and should be explored in future investigations.

Finally, the study was conducted over a 3-month period of supplementation, which may not fully capture the long-term effects of dietary ginger supplementation on liver function and redox balance. Longer-term studies and broader biomarker panels would strengthen understanding of the sustained impact and safety profile of ginger in aging models.

## 6 Conclusion

This study demonstrates that ginger dietary supplementation exerts significant dose- and age-dependent effects on liver antioxidant defense systems in mice. Our findings confirm the initial hypotheses regarding both the dose-dependency of ginger’s effects and their more pronounced effects in older animals for several key parameters. Ginger supplementation enhanced key markers of antioxidant capacity, including DPPH radical scavenging activity, reduced glutathione (GSH) levels, and total antioxidant capacity, particularly at higher doses and in older animals. These findings highlight ginger’s potential to mitigate age-associated oxidative stress and support hepatic antioxidant defenses.

The differential impacts of ginger supplementation observed in this study underscore the importance of tailoring dietary interventions based on age and dosage. The demonstrated dose-dependent effects emphasize the importance of appropriate dosing strategies, while age-specific responses highlight the need for personalized antioxidant supplementation strategies. Future research should focus on elucidating the molecular mechanisms underlying these age-specific effects and investigating the long-term implications of ginger supplementation on liver health across different life stages.

Overall, this study establishes ginger as a promising dietary supplement for supporting liver antioxidant defenses, particularly in aging populations, while also highlighting the complexity of its biological effects across different age groups. These findings contribute to our understanding of natural antioxidant interventions and their potential role in promoting healthy liver aging.

## Data Availability

The original contributions presented in the study are included in the article/supplementary material, further inquiries can be directed to the corresponding author.
